# Reply to the letter to the editor “beyond percutaneous endoscopic gastrostomy: a broader view of nutritional care in older adults “ by Ola Sultan, Benjamin HL Harris and Louis J Koizia regarding our publication “longitudinal trends of percutaneous endoscopic gastrostomy in geriatric hospital units in Germany “

**DOI:** 10.1007/s41999-026-01476-0

**Published:** 2026-04-14

**Authors:** Rainer Wirth

**Affiliations:** Department of Geriatric Medicine, Marien Hospital Herne–University Hospital of Ruhr-University Bochum, Hölkeskampring 40, 44625 Herne, Germany

**Keywords:** Gastrostomy, Geriatric, Malnutrition, Tube feeding

Dear Ola Sultan, Benjamin HL Harris and Louis J Koizia,

We appreciate your interest in our work and your support for the interpretation of our findings. As mentioned in your comment, we think that the publication of respective guideline recommendations may have led to a more selective indication, particularly excluding severe dementia, together with a shift to a broader repertoire of non-invasive nutritional interventions. We fully support all your additions including a possible shift in case-mix and a broader cultural and ethical shift in geriatric medicine, including a greater integration of shared decision-making.

However, this is a simple database analysis which could only analyse the few data that were available. These observational data do not allow to prove any causality with regard to the marked longitudinal reduction of PEG use in geriatrics. Therefore, we tried to be cautious with our interpretation. As you pointed out, feeding decisions are inherently complex and depend on many individual factors, reaching far beyond a pure medical indication. We are confident that the marked reduction in PEG use over time reflects a meaningful progress in geriatric medicine. However, if the low rate of PEG use is now adequate or even too low is impossible to determine without more detailed individual data and without randomized controlled trials in well-defined patient groups, which are still not available.

In conclusion, we think that clinicians using PEG in geriatric medicine during the last decades experienced that the supposedly simple solution of a PEG is rarely able to solve complex clinical problems accompanied by low nutritional intake. We hope that research in this area will progress and allow more precise and tailored evidence-based recommendations in the future.

Rainer Wirth on behalf of all co-authors.

